# The 3′ Non-Coding Sequence Negatively Regulates PD-L1 Expression, and Its Regulators Are Systematically Identified in Pan-Cancer

**DOI:** 10.3390/genes14081620

**Published:** 2023-08-13

**Authors:** Zike Chen, Hui Pi, Wen Zheng, Xiaohong Guo, Conglin Shi, Zhiyang Wang, Jie Zhang, Xuanhao Qu, Lehan Liu, Haoliang Shen, Yang Lu, Miaomiao Chen, Weibing Zhang, Rong Sun, Yihui Fan

**Affiliations:** 1 Laboratory of Medical Science, School of Medicine, Nantong University, Nantong 226001, China; 2031110148@stmail.ntu.edu.cn (Z.C.); 1910110206@stmail.ntu.edu.cn (Z.W.); vk7ujc@163.com (J.Z.); qxh011005@163.com (X.Q.); liukun@ntu.edu.cn (L.L.); chenmm1024@ntu.edu.cn (M.C.); 2 Department of Pathophysiology, School of Medicine, Nantong University, Nantong 226001, China; pihui514@163.com; 3 Department of Pathogenic Biology, School of Medicine, Nantong University, Nantong 226001, China; z0107w@163.com (W.Z.); gxh9606@163.com (X.G.); sclynne@163.com (C.S.); 4 The Intensive Care Unit, Affiliated Hospital of Nantong University, Nantong 226001, China; shenhaoliang2006@126.com (H.S.); marineluyang@ntu.edu.cn (Y.L.); 5 Nantong Center for Disease Control and Prevention, Nantong 226001, China; ntzwb71@163.com

**Keywords:** 3′-untranslated region (3′-UTR), PD-L1, RNA-binding proteins (RBPs), miRNAs, m6A regulator

## Abstract

The 3′-untranslated region (3′-UTR) of PD-L1 is significantly longer than the coding sequences (CDSs). However, its role and regulators have been little studied. We deleted whole 3′-UTR region by CRISPR-Cas9. Prognostic analysis was performed using online tools. Immune infiltration analysis was performed using the Timer and Xcell packages. Immunotherapy response prediction and Cox regression was performed using the R software. MicroRNA network analysis was conducted by the Cytoscape software. The level of PD-L1 was significantly and dramatically up-regulated in cells after deleting the 3′-UTR. Additionally, we discovered a panel of 43 RNA-binding proteins (RBPs) whose expression correlates with PD-L1 in the majority of cancer cell lines and tumor tissues. Among these RBPs, PARP14 is widely associated with immune checkpoints, the tumor microenvironment, and immune-infiltrating cells in various cancer types. We also identified 38 microRNAs whose individual expressions are associated with PD-L1 across different cancers. Notably, miR-3139, miR-4761, and miR-15a-5p showed significant associations with PD-L1 in most cancer types. Furthermore, we revealed 21 m6A regulators that strongly correlate with PD-L1. Importantly, by combining the identified RBP and m6A regulators, we established an immune signature consisting of RBMS1, QKI, ZC3HAV1, and RBM38. This signature can be used to predict the responsiveness of cancer patients to immune checkpoint blockade treatment. We demonstrated the critical role of the 3′-UTR in the regulation of PD-L1 and identified a significant number of potential PD-L1 regulators across various types of cancer. The biomarker signature generated from our findings shows promise in predicting patient prognosis. However, further biological investigation is necessary to explore the potential of these PD-L1 regulators.

## 1. Introduction

In various types of human cancers, immunotherapies that target the PD-1/PD-L1 axis have achieved remarkable success [1]. These immunotherapies involve the use of antibodies to block the interactions between the PD-1/PD-L1 immune checkpoints, which have proven to be highly effective in clinical settings [2]. Specifically, therapy with PD-L1 antibodies has demonstrated efficacy in blocking membrane PD-L1 and treating certain advanced carcinomas [3].

Immunotherapy offers significant advantages over other cancer treatments due to its involvement of the immune system [4]. In addition to being safer than traditional methods such as radiation and chemotherapy, it also establishes a lasting memory similar to antigens, which plays a crucial role in preventing tumor recurrences [5]. A FDA-approved immunohistochemistry test for PD-L1 expression is a prerequisite for treatment with anti-PD-1/PD-L1 therapy [6]. However, most tumors do not respond effectively to single-agent PD-1 antibodies, with only classic Hodgkin’s lymphoma showing effectiveness with PD-1 inhibitors alone [7]. Furthermore, approximately 5–10% of patients develop severe immune-related inflammatory reactions, and 15–35% of patients experience disease recurrence after a period of PD-1/PD-L1 monoclonal antibody use [7].

To address these questions, further understanding of the regulatory mechanism of PD-L1 expression is needed. The PD-L1 gene is constitutively expressed by a wide range of hematopoietic cells, such as macrophages, DCs, T cells, B cells, mast cells, and certain non-hematopoietic cells [8]. Additionally, PD-L1 can be upregulated in various cell types in response to inflammatory cytokines and other stimuli, and it is often overexpressed in cancer cells [9]. The regulation of PD-L1 expression involves exosome transport, post-transcriptional and post-translational modifications, and gene transcription [10,11]. Expanding our understanding of PD-L1 expression regulation is crucial for improving the efficacy of the current immune checkpoint blockade (ICB) and advancing cancer immunotherapy.

In our previous study, we discovered a fascinating phenomenon: the presence of a 2692 nt-long 3′-UTR region in a full-length 3634 nt PD-L1 mRNA molecule. In this study, we aimed to investigate the regulatory role of this lengthy 3′ non-coding region in PD-L1 expression and understand the underlying mechanism through bioinformatics research methods. Additionally, we explored the potential clinical implications of our findings.

## 2. Material and Method

### 2.1. Cell Culture and Treatment

Human breast cancer cells (SUM-159 (RRID: CVCL_5423)) were purchased from Cobioer; Nanjing Kebai Biotechnology Co., Ltd., Nanjing, China. The cells were cultured in DMEM supplemented with 10% FBS, 100 U/mL penicillin, and 100 mg/mL streptomycin. They were cultured in a humidified atmosphere of 5% CO_2_ at 37 °C. All cells went through mycoplasma detection and were free of mycoplasma. The cells were inoculated in six-well plates, and the cells were seeded at the same density and allowed to adhere for 24 h before treatment. All human cell lines have been authenticated using STR profiling within the last 3 years, and all experiments were performed with mycoplasma-free cells.

### 2.2. Plasmids

We constructed sgPD-L1-A1 (5′-GGAACTTCTGATCTTCAAGC-3′), sgPD-L1-A2 (5′-AAGATCAGAAGTTCCAATGC-3′), sgPD-L1-E1(5′-GTCAATGACAAGGAGTACCT-3′), and sgPD-L1-E2 (5′-GATAACACAAGGAGCTCTGT-3′) by ligation and digestion. The targeting oligonucleotides were cloned into the epiCRISPR vector. The vector plasmids were treated with BSPQI enzyme (New England Biolads, Ipswich, MA, USA) at 50 °C for 4 h. The upstream and downstream primers were annealed at 94 °C for 20 min then cooled at room temperature for one hour. The ligation was performed by using T4 DNA ligase (New England Biolads) at 16 °C overnight. The plasmids as mentioned above were validated by DNA sequencing.

### 2.3. Establishment of Stable Cell Lines

To construct a stable PD-L1-3′-UTR knockout cell line, SUM-159 cells were co-transfected with a combination of designed sgRNAs and transfected by the epiCRISPR vector plasmid as the control using Lipo2000 (Invitrogen, Waltham, MA, USA). After 48 h of transfection, the medium was replaced by complete medium containing 2 μg/mL puromycin (InvivoGen, San Diego, CA, USA) for 7 days to select stable cell lines. The stable cell line was confirmed by genotyping. The cells were lysed by 50 μL 1x mouse tissue lysis buffer (Vazyme) with 1 μL protease K (Vazyme) at 55 °C for 30 min. Then, the genomic PCR was performed with2xTaq Plus Master Mix (Dye Plus) (Vazyme) under the manufacturer’s instructions. The PCR products were analyzed with agarose gel electrophoresis. The DNA was purified for DNA sequencing, and the DNA sequencing was performed by Suzhou Anshengda Company, Suzhou, China.

### 2.4. RNA Isolation and Quantitative Real-Time PCR

Total RNA was extracted from the cells by using TRIzol RNA isolation reagents (Invitrogen). The purity and concentration of total RNA were evaluated using a Nanodrop 1000 spectrophotometer. Isolated RNA was then reverse-transcribed to cDNA according to the instructions of the HiScript^®^ II QRT SuperMix for qPCR (+gDNA wiper) kit. The expression of the targeted gene was detected by the AceQ^®^ qPCR SYBR Green Master Mix kit (Vazyme, Nanjing, China) according to the confirmed instructions using the CFX96 real-time PCR system (Bio Rad, Hercules, CA, USA). Fold changes in gene expression were calculated using the 2^−△△t^ method and normalized to the expression of GAPDH.

### 2.5. Western Blot Analysis

Cells were collected and lysed by RIPA buffer (Solarbio, Beijing, China) containing 1% PMSF (Solarbio). SDS-PAGE was performed according to the manufacturer’s instructions. PVDF membranes (Millipore, Burlington, MA, USA) were used to transfer the proteins from the gels. Then, these were blocked in 5% non-fat milk in TBST for 2 h at room temperature. After blocking, the membranes were incubated with the following primary antibodies including anti-PD-L1 (Abcam, Cambridge, UK, 1:1000) and anti-GAPDH (Santa Cruz Biotechnology, Dallas, TX, USA, 1:2000) overnight at 4 °C. After incubation with horseradish peroxidase-conjugated secondary antibodies for 2 h at room temperature, the blots were visualized using an ECL kit (BL520B; Biosharp, Sakai, Japan). Finally, the images were analyzed using the ImageJ software.

### 2.6. Cellular Immunofluorescence

In advance, the cells were plated and allowed to climb on round glass slides in 24-well plate at the same density. After 24 h, the slides of the cells were fixed with 4% paraformaldehyde (PFA) for 20 min at room temperature, and then, the cells were washed with PBS (3 times for 5 min each), then blocked with 1% BSA for 2 h at room temperature. Then, the primary antibody including anti-PD-L1 (Abcam, 1:200) was incubated with the samples at 4 °C overnight. After washing 3 times for 5 min each, the secondary antibody, was Alexa-Fluor-488-conjugated anti-rabbit (Beyotime, Shanghai, China, 1:500), was added and incubated overnight. Care was taken to avoid light after adding the secondary antibody. After PBS washing (3 times for 5 min each), Hoechst (Beyotime, 1:500) was used for nuclear staining. The confocal microscope (Leica, Wetzlar, Germany) was applied to examine the fluorescence of stained cells.

### 2.7. Flow Cytometry

Cells were seeded into 6-well plates at the same density. The next day, the cells were collected and washed with PBS 3 times and stained with antibodies including anti-PD-L1 (PE-conjugated) (Elabscience, Houston, TX, USA, E-AB-F1133D) and anti-PD-L2 (APC-conjugated) (Elabscience, Houston, TX, USA, E-AB-F1175E) in staining buffer for 40 min on ice. During this step, light was avoided. After that, the samples were centrifuged for 3 min and washed 3 times, then suspended with PBS. The signals were captured by a BD Calibur (BD Biosciences, San Jose, CA, USA) flow cytometer and analyzed by the FlowJo software.

### 2.8. Data Acquisition

Gene expression TPM values of the protein-coding genes of 1406 Cancer Cell Lines were downloaded from the Broad Institute Cancer Cell Line Encyclopedia (CCLE) database (https://sites.broadinstitute.org/ccle/, accessed on 5 December 2022). Transcriptome RNA-seq, miRNA-seq of 1,0327 patients with the 33 most-common cancers (adrenocortical carcinoma (ACC), bladder carcinoma (BLCA), breast invasive carcinoma (BRCA), cervical squamous cell carcinoma and endocervical adenocarcinoma (CESC), cholangiocarcinoma (CHOL), colon adenocarcinoma (COAD), lymphoid neoplasm diffuse large B cell lymphoma (DLBC), esophageal carcinoma (ESCA), glioblastoma multiforme (GBM), head and neck squamous cell carcinoma (HNSC), kidney chromophobe (KICH), kidney renal clear cell carcinoma (KIRC), kidney renal papillary cell carcinoma (KIRP), acute myeloid leukemia (LAML), brain lower-grade glioma (LGG), liver hepatocellular carcinoma (LIHC), lung adenocarcinoma (LUAD), lung squamous cell carcinoma (LUSC), mesothelioma (MESO), ovarian serous cystadenocarcinoma (OV), pancreatic adenocarcinoma (PAAD), pheochromocytoma and paraganglioma (PCPG), prostate adenocarcinoma (PRAD), rectum adenocarcinoma (READ), sarcoma (SARC), skin cutaneous melanoma (SKCM), stomach adenocarcinoma (STAD), testicular germ cell tumors (TGCT), thyroid carcinoma (THCA), thymoma (THYM), uterine corpus endometrial carcinoma (UCEC), uterine carcinosarcoma (UCS), and uveal melanoma (UVM)) were downloaded from The UCSC Xena database (https://xenabrowser.net/, accessed on 12 April 2022). The corresponding clinicopathological information of 1,0327 patients were downloaded from The Cancer Genome Atlas (TCGA) database (https://portal.gdc.cancer.gov/, accessed on 18 April 2022) IMvigor210 is a multicenter, single-arm, phase II trial to assess the safety and efficacy of atezolizumab (a PD-L1 inhibitor) in patients with locally advanced and metastatic urothelial carcinoma. After procuring the Creative Commons 3.0 License, we obtained the transcriptome RNA sequencing (RNA-seq) and detailed clinical annotations from IMvigor210 Core Biologies (http://research-pub.gene.com/IMvigor210CoreBiologies/, accessed on 10 October 2022).

### 2.9. Prognostic Analysis

We acquired the overall survival map data of RBP and m6A-related genes using GEPIA2 (http://gepia2.cancer-pku.cn/#analysis/, accessed on 20 January 2023). Besides, we obtained the overall survival map data of microRNA using ENCORI Starbase (https://starbase.sysu.edu.cn/index.php/, accessed on 20 September 2022).

### 2.10. Immune Microenvironment and Immune Cell Infiltration Analysis

The data to evaluate the correlation between gene expression and the content of immune and stromal cells in tumor tissues were obtained from Sanger box web (http://sangerbox.com/, accessed on 30 October 2022) through the Timer and Xcell packages.

### 2.11. Immunotherapy Response Prediction

Tumor immune dysfunction and exclusion (TIDE) (http://tide.dfci.harvard.edu/, accessed on 10 January 2023) is a computational framework developed to evaluate the potential of tumor immune escape from the gene expression profiles of cancer samples. The TIDE score computed for each tumor sample can serve as a surrogate biomarker to predict the response to immune checkpoint blockade for multiple types of cancers.

### 2.12. Statistical Analysis

Statistical analyses were conducted using the R software (Version 4.2.1). Continuous variables are presented as the standard error of the mean and were compared using Student’s *t*-test or the Wilcoxon rank sum test. Figure panels were pieced together by Adobe Illustrator (CC 2018). Some figures were drawn using the R software (Version 4.2.1), Tbtools (Version 1.06), and Figdraw (https://www.figdraw.com/static/index.html/, accessed on 27 December 2022). Univariate and multivariate Cox proportional hazards regression analysis was performed using the “survival” package. A least absolute shrinkage and selection operator (LASSO) regression model was performed with the “glmnet” and “survival” packages. Kaplan–Meier survival analysis with the log-rank test was performed with the R package “survminer”. Statistical significance was set at *p* < 0.05 and is shown as * *p* < 0.05, ** *p* < 0.01, and *** *p* < 0.001.

### 2.13. MicroRNA Network Analysis

MicroRNA network analysis was conducted using the Cytoscape software (Cytoscape, 3.9.1).

## 3. Results

### 3.1. The 3′-Untranslated Region Strongly Restricts PD-L1 Expression

**Figure 1:**
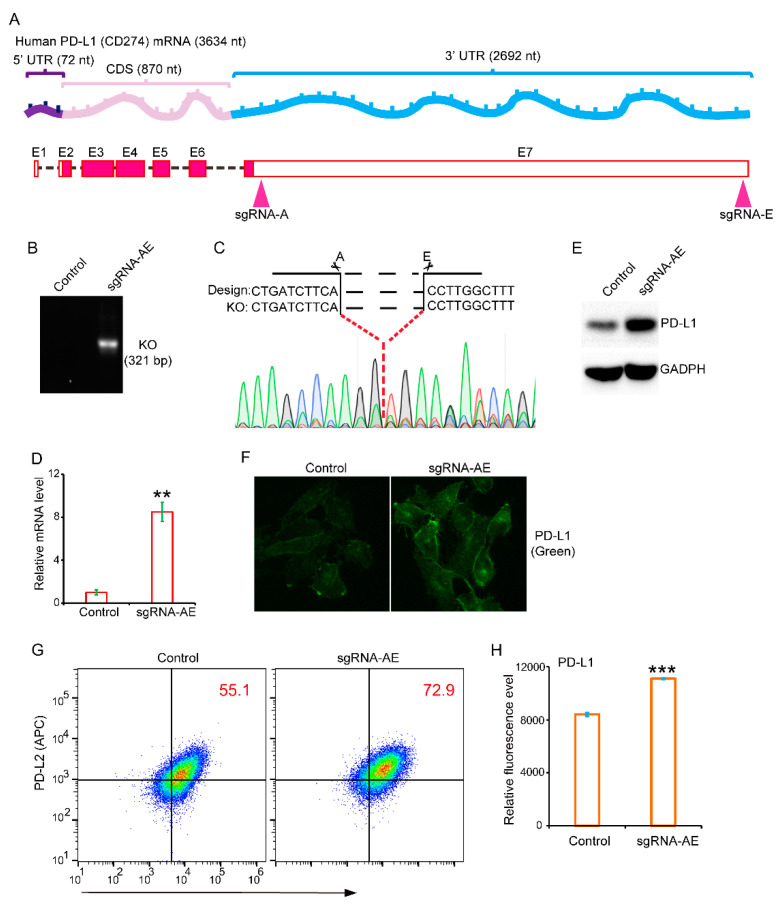
Deletion of whole 3′-UTR and the effect on PD-L1 expression. (**A**) Schematic presentation of PD-L1 mRNA and its 3′-UTR. The location of sgRNAs for CRISPR/Cas9-mediated deletion is indicated. (**B**) Genomic PCR was used to determine the deletion of 3′-UTR. (**C**) The DNA sequence of the joint region after deletion of 3′-UTR is shown. (**D**) RT-PCR was used to determine the mRNA level of control and 3′-UTR-deficient cells (sgRNA-AE). (**E**) WB was used to determine the protein level of control and 3′-UTR-deficient cells. (**F**) Cellular immunofluorescence was used to examine the PD-L1 distribution and level in control and 3′-UTR-deficient cells. (**G**,**H**) Flow cytometry was used to measure the surface expression of PD-L1 in control and 3′-UTR-deficient cells. ** *p*-value < 0.01, *** *p*-value < 0.001.

The messenger RNA (mRNA) of PD-L1 in humans is 3634 nucleotides (nt) long. Within this length, the coding sequence spans 870 nt, while the 3′-untranslated region measures 2692 nt (Figure 1A). To investigate the role of the 3′-UTR, we designed two sgRNAs to delete it in cells (Figure 1A). Subsequently, we conducted PCR to examine the genotype after establishing stable cells. In knockout cells, we obtained the expected PCR product, and DNA sequencing clearly demonstrated the deletion of the 3′-UTR (Figure 1B,C). Next, we assessed the mRNA level through RT-PCR in both control and 3′-UTR-deficient cells. As shown, the level of mRNA in 3′-UTR-deficient cells was significantly higher than in control cells (Figure 1D). To further confirm our findings, we conducted WB to examine the protein level of PD-L1. As expected, the protein level of PD-L1 in 3′-UTR-deficient cells was significantly higher than in control cells (Figure 1E). Cellular immunofluorescence also showed the significantly increased PD-L1 level in 3′-UTR-deficient cells (Figure 1F). Flow cytometry analysis further supported these findings, showing a significantly enhanced surface level of PD-L1 in 3′-UTR-deficient cells (Figure 1G,H). Collectively, our results clearly demonstrated the critical negative role of the 3′-UTR in restricting the expression of PD-L1 in cancer cells.

### 3.2. Identification of a Panel of 43 RNA-Binding Proteins That Correlated with PD-L1 in a Majority of Cancer Cell Lines and Tumor Tissues

**Figure 2:**
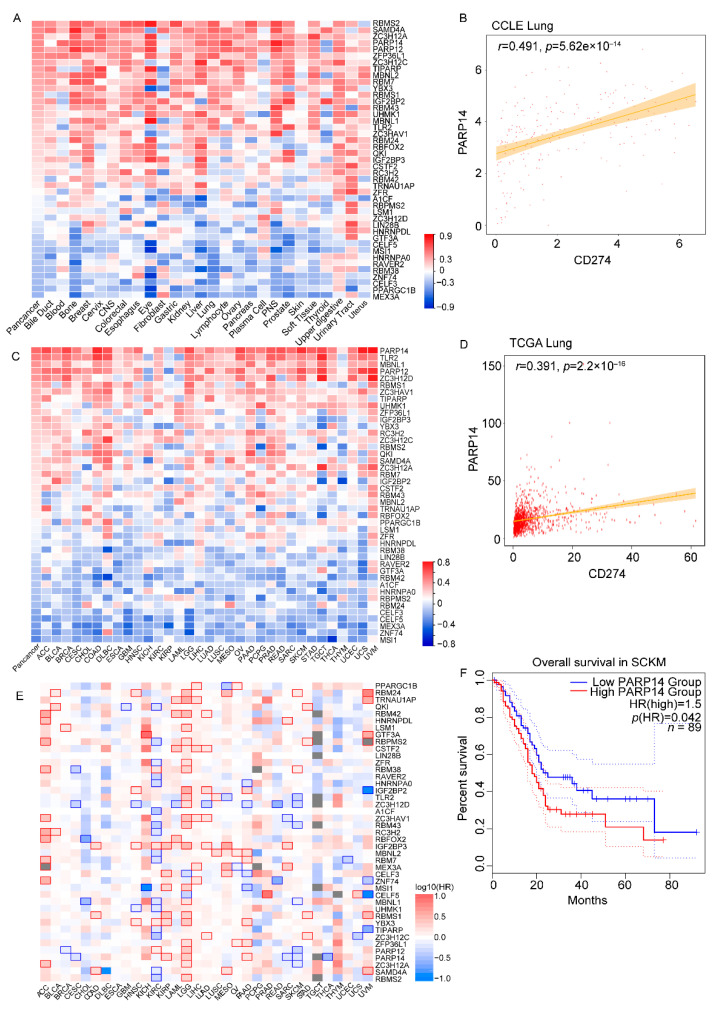
PD-L1-expression-related RBP screening, and RBP prognosis analysis in the TCGA cohort and cancer cell lines. (**A**) Correlation analysis of gene expression profiles between PD-L1 and 408 RBPs in 1406 cancer cell lines from Cancer Cell Line Encyclopedia (CCLE). The expression of PD-L1 was significantly associated with 43 RBP in pan-cancer. (**B**) Positive correlation between PARP14 expression and PD-L1 expression in lung cancer cell lines. (**C**) Correlation analysis of gene expression profiles between PD-L1 and 43 RBPs in tumor tissue from pan-cancer RNA-seq data of The Cancer Genome Atlas (TCGA) database. (**D**) Positive correlation between PARP14 expression and PD-L1 expression in the TCGA cohort. The Spearman *r*-values and *p*-values are shown in the scatter plots. (**E**) Survival map of 43 RBPs from 33 types of tumors using GEPIA2. The blue square indicates that low expression of the indicated gene was correlated with poor survival of patients (*p* < 0.05), and the red square indicates that high expression of the indicated gene was correlated with poor survival of patients (*p* < 0.05). (**F**) Kaplan–Meier plot of the overall survival of patients with SKCM based on PARP14 expression levels from the TCGA data at a cutoff of 50%.

Due to the critical role of the 3′-UTR in the regulation of PD-L1, we conducted a systematic study to identify its regulators. One of the most-extensively studied proteins that regulate gene expression through 3′-UTR are RNA-binding proteins (RBPs). Therefore, we initially examined the mRNA levels of PD-L1 and 408 RBPs in a panel of cancer cell lines (1406 cell lines) from the Cancer Cell Line Encyclopedia (CCLE). Our findings revealed that 43 RBPs showed a strong correlation with PD-L1 (absolute correlation score > 0.2 and *p*-value < 0.05) (Figure 2A). Among these 43 RBPs, the majority exhibited a positive correlation with PD-L1. For instance, the expression level of PARP14 positively correlated with PD-L1 in lung cancer cell lines (*r* = 0.491, *p*-value = 5.62 × 10^−14^) (Figure 2B). Subsequently, we verified the correlation between these 43 RBPs and PD-L1 in the TCGA database, obtaining similar results (Figure 2C). In addition, we found a positive correlation between PARP14 expression level and PD-L1 in lung cancer (LUAD + LUSC) (R = 0.391 *p*-value < 2.2 × 10^−16^) (Figure 2D). To uncover the role of the identified RBPs in cancer, we utilized the GEPIA2 web server, which analyzes data from 33 different cancer types. We observed a significant correlation between RBP expression and prognosis in multiple cancers, using a cutoff of a 50% high expression threshold (Figure 2E). Notably, high levels of PARP14 expression were associated with poorer overall survival (OS) in PAAD (*p*-value = 0.00013) (Figure 2F). Taken together, we identified 43 PD-L1-associated RBPs, and the majority of them are associated with patient prognosis.

### 3.3. PARP14 Is Widely Associated with Immune Checkpoints, Tumor Microenvironment, and Immune-Infiltrating Cells in Various Cancer Types

**Figure 3:**
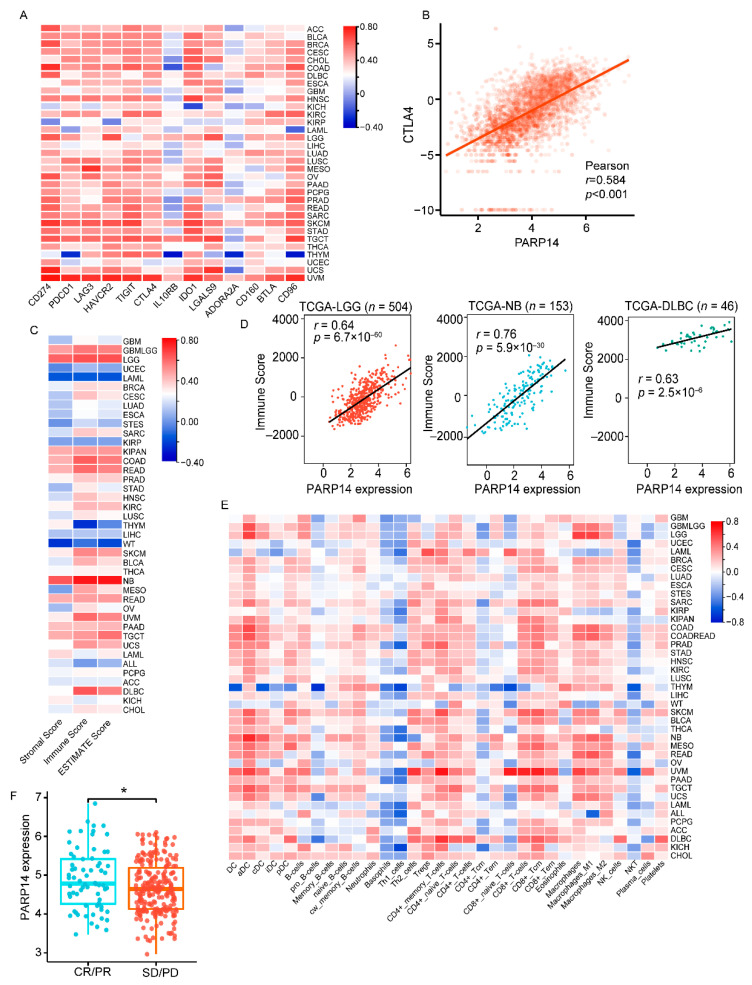
Immuno-infiltration analysis in tissues with distinct expression of PARP14. (**A**) Correlation analysis of gene expression profiles between PARP14 and immune checkpoints (ICs) in tumors from pan-cancer clinical data of the TCGA database. (**B**) Positive correlation between PARP14 expression and CTLA4 expression from the data of TCGA. The Spearman *r*-values and *p*-values are shown in the scatter plots. (**C**) Correlation between StromalScore, ImmuneScore, EstimateScore, and PARP14 across different cancer types via the Sanger box tool. There was a significantly positive correlation in most tumor types. (**D**) The correlation between PARP14 expression and ImmuneScore in three cancers (NB, LGG, and DLBC). The Pearson *r*-values and *p*-values are shown in the scatter plots. (**E**) Xcell analysis of the correlation between different immune cell infiltration and PARP14 expression in different cancer types via Sanger box tools. (**F**) The expression of PARP14 between the complete response (CR)/ partial response (PR) group and the stable disease (SD)/ progressive disease (PD) group in the imvigor 210 cohort was analyzed via the *t*-test. * *p* < 0.05.

To further explore the importance of our identified RBPs, we conducted a detailed analysis on PARP14 as an example. Firstly, we investigated the correlation between PARP14 and other immune checkpoints (ICs) in pan-cancer data from TCGA. Surprisingly, we found that the expression of PARP14 was positively correlated with most immune checkpoints, including PDCD1, LAG3, HAVCR2, TIGIT, CTLA4, IL10RB, IDO1, LGALS9, ADORA2A, BTLA, and CD96 (Figure 3A). Notably, we observed a strong correlation between PARP14 and CTLA-4 (*r* = 0.584, *p* < 0.01) (Figure 3B).

In order to explore the role of PARP14 in the tumor immunity of patients with various cancers, we calculated three ESTIMATE indices in each sample to evaluate the proportions of stromal and immune cells (Figure 3C). The results showed that PARP14 was positively correlated with immune infiltration in 26 cancers with *r*-values greater than 0.3. We present representative examples of the three tumors with the highest immune infiltration in Figure 3D. Furthermore, we utilized the Xcell algorithm to examine the correlation between PARP14 and 32 immunoinfiltrating cells in 41 different types of cancer. In our study, we found that most infiltrative immune cells exhibited a positive correlation with PARP14, while the infiltration levels of Basophils, Th1_cells, and NKT cells showed a significant negative correlation with PARP14 (Figure 3E). To investigate the role of PARP14 in response to immunotherapy, we conducted a comparison of PARP14 expression levels between two groups: the complete response/part response (CR/PR) group and the stable disease/progressive disease (SD/PD) group in the Imvigor 210 cohort. The results revealed that the PARP14 mRNA levels were significantly higher in the immunotherapy CR/PR group compared to the SD/PD group (*p*-value < 0.05) (Figure 3F). Hence, our findings provide evidence for the crucial involvement of the identified RBPs and emphasize the critical role of PARP14 in regulating cancer immunity. Additionally, our results suggest the potential utility of PARP14 as a biomarker for immune response. 

### 3.4. Comprehensively Identification of 38 MicroRNAs Associated with PD-L1 in a Majority of Tumor Tissues

**Figure 4:**
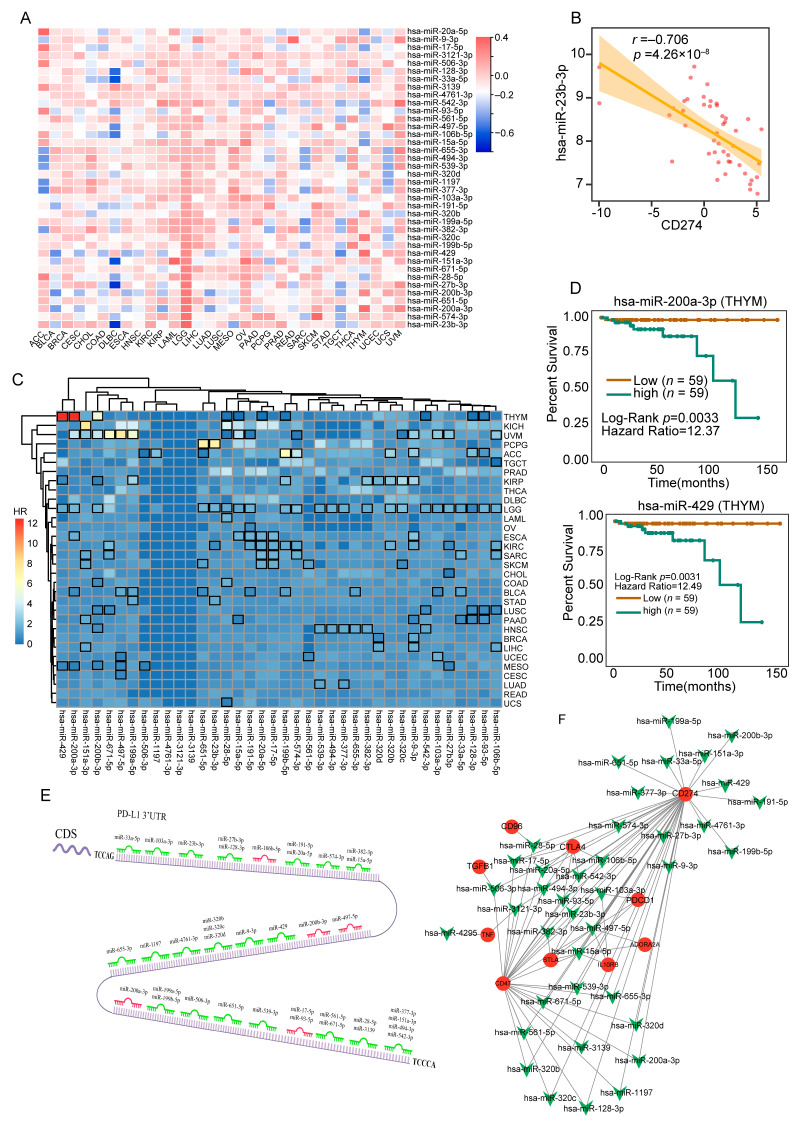
Analysis of potential miRNAs regulating PD-L1 expression. (**A**) Correlation analysis of expression profiles between PD-L1 and 38 miRNAs predicted by ENCORI tools in different tumor types from the data of TCGA. (**B**) Positive correlation between has-miR-23b-3p expression and PD-L1 expression in TGCT from TCGA. (**C**) The overall survival analysis based on the expression status of 38 miRNAs in 32 types of tumors from ENCORI (https://starbase.sysu.edu.cn/, accessed on 20 September 2022). The heatmap represents the risks ratio (HR) of each miRNA in different tumors, where the black box marks the *p*-value less than 0.05 for the overall survival analysis. (**D**) Survival curve for KIRC patients with different miRNAs’ expression. Patients were divided into two groups according to the expression of hsa-miR-200a-3p (up) and hsa-miR-429 (bottom). (**E**) Potential binding sites and sequences for the identified microRNAs significantly associated with PD-L1. MicroRNAs that have already been reported in the articles are highlighted in red, and those not reported are highlighted in green. (**F**) Network diagram of the ceRNA network involving PD-L1 and other immune checkpoints using starBase. The red circles represent PD-L1 and other immune checkpoints, and the green arrows represent different miRNAs.

miRNAs play a crucial role in regulating gene expression through the 3′-UTR. In this study, we aimed to investigate the microRNAs that can potentially influence the expression of PD-L1. Firstly, we used the starBase website to predict potential PD-L1-binding miRNAs and identified 108 potential microRNAs. Subsequently, we examined the correlation between mRNA levels of PD-L1 and these 108 microRNAs in the TCGA database. Then, we screened for miRNAs that exhibited a significant negative association with PD-L1 expression in at least 20 tumors. We found that 38 miRNAs showed a strong negative correlation with PD-L1 (Figure 4A). For instance, hsa-miR-23b-3p expression negatively correlated with PD-L1 in DLBC (*r* = −0.706, *p*-value = 4.26 × 10^−8^) (Figure 4B). Next, we explored the prognostic value of the identified miRNAs in different cancer patients (Figure 4C). Kaplan–Meier analysis showed that THYM patients with a high expression of hsa-miR-200a-3p and hsa-miR-429 had significantly better overall survival compared to patients with low expression of hsa-miR-200a-3p (*p*-value = 0.0033) and hsa-miR-429 (*p*-value = 0.0031) (Figure 4D). Figure 4E displays the potential binding sites of all 38 miRNAs, with the known PD-L1-associated miRNAs highlighted in red. There is a growing body of evidence suggesting that miRNA accessibility regulated by competitive endogenous RNA (ceRNA) plays a critical role in gene function. Taking this into consideration, we performed an analysis and constructed a ceRNA network involving PD-L1 and other immune checkpoints using miRNA Cancer Map (Figure 4F). Based on database mining, we constructed an immune checkpoint–miRNA interaction network. Among the 38 miRNAs analyzed, all 38 were found to interact with PD-L1. Additionally, 3 miRNAs interacted with IL10RB, 2 interacted with ADORA2A, 5 interacted with BTLA, 1 interacted with CD96, 6 interacted with CTLA4, 1 interacted with TGFB1, 2 interacted with TNF, 25 interacted with CD47, and 3 interacted with PDCD1. In total, we identified a set of 38 miRNAs that were highly negatively associated with PD-L1 and found that these miRNAs were associated with patient survival. 

### 3.5. Comprehensive Analysis of m6A Regulators in the Control of PD-L1 Expression

**Figure 5:**
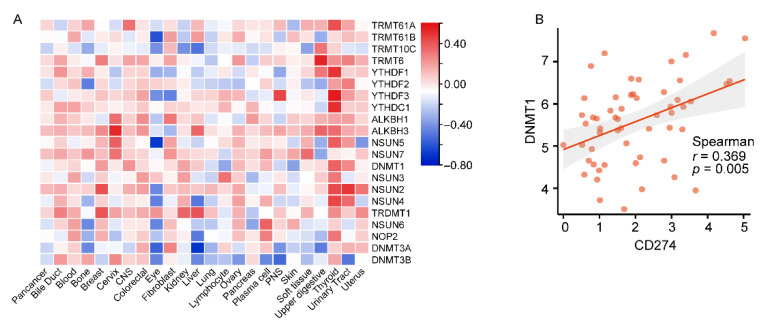

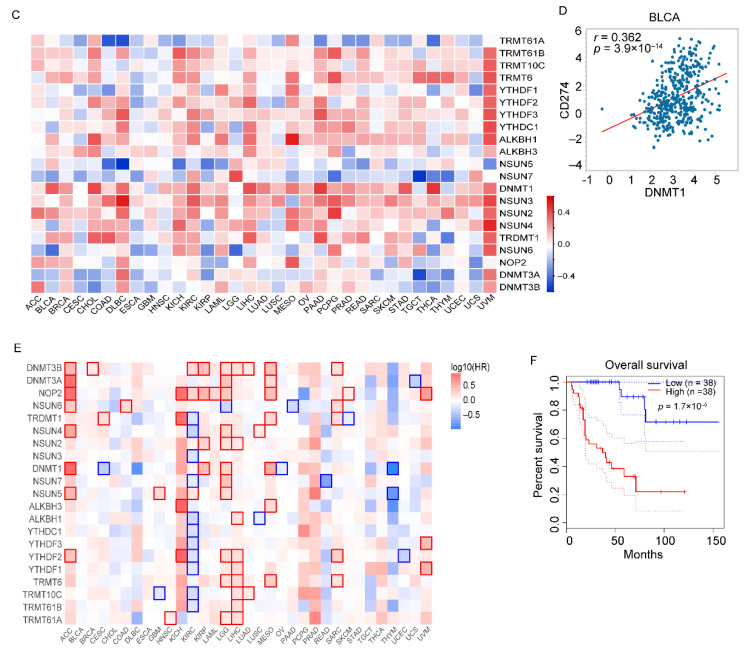
PD-L1-expression-related immune-related m6A regulators’ screening and immune-related m6A regulators’ prognosis analysis in the TCGA cohort. (**A**) Correlation analysis of gene expression profiles between PD-L1 and 21 m6A regulators in 1406 cancer cell lines from Cancer Cell Line Encyclopedia (CCLE). (**B**) Positive correlation between DNMT1 expression and PD-L1 expression in upper aerodigestive cancer cell lines. The Spearman *r*-values and *p*-values are shown in the scatter plots. (**C**) Correlation analysis of gene expression profiles between PD-L1 and 21 m6A regulators in 33 tumor tissue from the pan-cancer RNA-seq data of The Cancer Genome Atlas (TCGA) database. (**D**) Positive correlation between DNMT1 expression and PD-L1 expression in BLCA. The Spearman *r*-values and *p*-values are shown in the scatter plots. (**E**) Survival map of 21 m6A regulators from 33 types of tumors using GEPIA2. The blue square indicates that low expression of the indicated gene was correlated with the poor survival of patients (*p* < 0.05), and the red square indicates that high expression of the indicated gene was correlated with the poor survival of patients (*p* < 0.05). (**F**) Kaplan–Meier plot of the overall survival of patients with ACC based on DNMT1 expression levels from the TCGA data at a cutoff of 50%.

**Figure 6:**
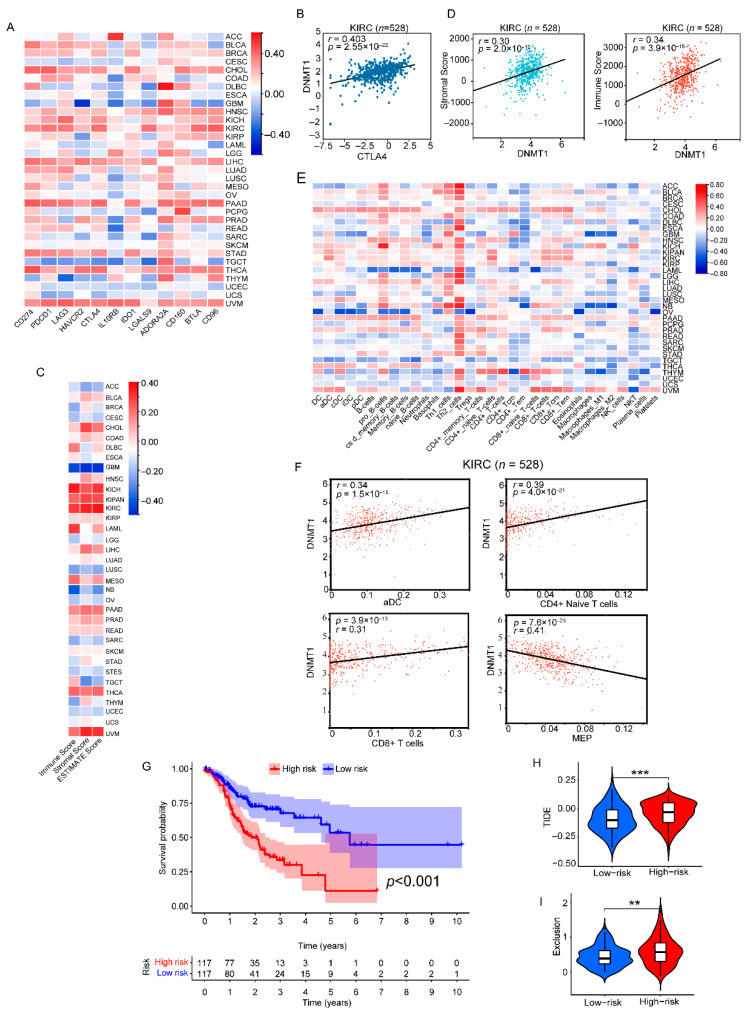
Correlation analysis of DNMT1 with immune checkpoint genes, the tumor microenvironment, and immune-infiltrating cells and LASSO-Cox regression model building based on 43 RBP genes and 21 m6A-related genes in STAD to select candidate variables associated with OS. (**A**) Correlation analysis of gene expression profiles between DNMT1 and immune checkpoints (ICs) in tumors from pan-cancer clinical data of the TCGA database. (**B**) Positive correlation between DNMT1 expression and CTLA4 expression in KIRC. The Spearman *r*-values and *p*-values are shown in the scatter plots. (**C**) Correlation between StromalScore, ImmuneScore, EstimateScore, and DNMT1 across 35 cancer types via the Sanger box tool. There was a significantly positive correlation in most tumor types. (**D**) The correlation between DNMT1 expression and StromalScore and ImmuneScore in KIRC. The Pearson *r*-values and *p*-values are shown in the scatter plots. (**E**) Xcell analysis of the correlation between different immune cell infiltration and DNMT1 expression in 35 cancer types via Sanger box tools. (**F**) The correlation between DNMT1 expression and aDC, CD4+ naive T cells, CD8+ T cells, and MEP cells in KIRC. The Pearson *r*-values and *p*-values are shown in the scatter plots. (**G**) Kaplan–Meier curves of the group showing OS in the low- and high-risk groups classified based on the median risk score. (**H**,**I**) Evaluation of the efficiency of ICI therapy by the TIDE score and Exclusion score between the high- and low-risk group. ** *p*-value < 0.01, *** *p*-value < 0.001.

Furthermore, another mechanism involved in the regulation of 3′-UTR is through m6A modification. To investigate this, we initially examined the mRNA levels of the PD-L1 and 68 m6A regulators in a panel of cancer cell lines. Our findings revealed a strong correlation between 21 immune-related m6A regulators and PD-L1 (Figure 5A). For example, the DNMT1 expression level positively correlated with PD-L1 in upper aerodigestive cancer cell lines (*r* = 0.369, *p*-value = 0.005) (Figure 5B). We then validated this correlation between the 21 m6A regulators and PD-L1 using the TCGA database, obtaining similar results (Figure 5C). Specifically, the DNMT1 expression level was found to positively correlate with PD-L1 in BLCA (*r* = 0.362, *p*-value < 3.90 × 10^−14^) (Figure 5D). To further explore the impact of these m6A regulators in cancer, we mined public cancer gene expression and prognosis databases using the Gene Expression Profiling Interactive Analysis 2 (GEPIA2). Remarkably, across 33 different cancer types, we observed a significant correlation between m6A regulators and prognosis in several cancers (Figure 5E). In the case of DNMT1, higher levels of its expression were found to be associated with poorer overall survival (OS) for ACC (*p*-value = 1.7 × 10^−5^) (Figure 5F). Additionally, we examined the correlation between DNMT1 and other immune checkpoints (ICs) across various types of cancer. Our analysis revealed a significant correlation (*p* < 0.05 between DNMT1 and ADORA2A, IL10RB, and CTLA-4 in certain cancers (Figure 6A). For instance, DNMT1 exhibited a positive correlation with CTLA-4 (*r* = 0.403, *p*-value = 2.55 × 10^−22^) (Figure 6B). In order to explore the role of DNMT1 in tumor immunity among patients with different cancers, we calculated three indices of ESTIMATE for each sample to assess the fractions of stromal and immune cells (Figure 6C). As depicted, DNMT1 demonstrated a positive correlation with the immunoscore and stromal score in KIRC (Figure 6D). 

The correlation between DNMT1 and 32 immunoinfiltrating cells in 33 different types of cancers was investigated using the Xcell algorithm. Our study revealed a positive correlation between Th2_cells and DNMT1 in various cancer types (Figure 6E). Importantly, DNMT1 exhibited a significant positive correlation with Th2_cell content in all tumors. In the case of KIRC, DNMT1 expression was found to be positively correlated with DC, naive T cells, and MEP cell content (Figure 6F), indicating a potential association between DNMT1 expression and immune infiltration by these specific immune cell types in KIRC.

### 3.6. Combination of Different PD-L1-3′-UTR Regulators Allowed Us to Establish The Predictive Immune Signature (RBMS1, QKI, ZC3HAV1, RBM38) in STAD

Although immune checkpoint blockade is effective in 20% of cancer patients, it is largely unpredictive. Due to the critical role of the 3′-UTR in the regulation of PD-L1 and the identification of various layers of PD-L1 regulators, we tried to explore a signature to potentially indicate the response of ICB. We performed LASSO-Cox regression analysis on the identified 43 RBP genes and 21 m6A-related genes to construct a risk score for the prediction of overall survival and the efficiency of immune checkpoint inhibitor (ICI) therapy (Figure S1A,B). Luckily, we identified that RBMS1(HR: 1.466, 95% CI: 1.065–2.017, *p*-value = 0.019) and QKI (HR: 1.529, 95% CI: 1.002–2.335, *p*-value = 0.049) are risk factors, and the other four genes YTHDC1 (HR: 0.525, 95% CI: 0.279–0.986, *p*-value = 0.045), ZC3HAV1, RBM38, and PPARGC1B were protective factors (Figure S1C). Using the minimum lambda criteria, six genes were selected for the construction of our model by multifactorial Cox regression (Figure S1A,B). Four genes (RBMS1, ZC3HAV1, QKI, RBM38) were finally obtained to construct a patient-risk-prediction model (Table S1). The patients are categorized into the high-risk and low-risk groups based on the median risk score. The formula is as follows:

$$Riskscore=EXP(5.17+(0.324\;*\;{exp}_{RBMS1}-1.07*{exp}_{ZC3HAV1}+0.487\;*\;{exp}_{QKI}-0.324\;*\;{exp}_{RBM38}))$$

EXP: the exponent of the natural constant e; exp_RBMS1_: the RBMS1 expression value corresponding to the sample; exp_ZC3HAV1_: the ZC3HAV1 expression value corresponding to the sample; exp_QKI_: the QKI expression value corresponding to the sample; exp_RBM38_: the RBM38 expression value corresponding to the sample.

In the TCGA cohort, 234 patients were categorized into the high- and low-risk subgroups based on the median value of their risk score. It was observed that patients with a higher risk score had a lower overall survival rate (Figure 6G). Interestingly, the TIDE score can serve as a reliable indicator of the effectiveness of ICI therapy (anti-PD1 and anti-CTLA4) (Figure 6H,I). A higher TIDE score declares a greater likelihood of immune escape and a lower reactivity to ICI therapy. In our study, the TIDE score and Exclusion score were higher in the high-risk group (Figure 6H,I). These findings suggest that patients in the high-risk group may exhibit poor responses to ICI therapy. 

## 4. Discussion

It is notable that PD-L1 has an unusually long 3′-UTR. In leukemia and lymphoma, some cancer cells delete the 3′-UTR to increase the expression of PD-L1 and evade immune surveillance [12]. However, genetic abnormalities in the PD-L1-3′-UTR are rarely observed in solid cancers. The overall impact of the 3′-UTR on PD-L1 expression in solid cancers has not been extensively studied. Here, we genetically deleted the entire 3′-UTR in cells with high PD-L1 expression. Surprisingly, even in a background of high PD-L1 expression, the disruption of the 3′-UTR still led to a significant and substantial increase in PD-L1 expression. These results suggest an extraordinary impact of the 3′-UTR on the expression of PD-L1. Because the genetic abnormalities of the 3′-UTR in solid tumor are very rare, it is likely that dysregulation of regulators in this region is a major pathway for increasing PD-L1 expression. This inference was partially supported by the fact that many PD-L1-RBPs, -miRNAs, and -m6A modifiers are dysregulated in cancer. Furthermore, these dysregulated regulators are known to be associated with prognosis and tumor immunity. In summary, this study provided evidence to support the notion that the 3′-UTR region plays a profound and negative role in regulating PD-L1 expression.

RNA-binding proteins (RBPs) play a crucial role in regulating spatiotemporal gene expression [13,14]. They are involved in all processes of RNA metabolism such as RNA splicing, polyadenylation, mRNA stability, and mRNA localization [15]. The 3′-UTR acts as a hub for post-transcriptional control by recruiting RBPs [16,17]. Here, we explored the relationship between RBPs and PD-L1 in cancer cell lines and tissues. This investigation allowed us to identify 43 RBPs that are significantly associated with PD-L1 in a wide range of cancer. Among these identified RBPs, RBMS2 and RBMS1 are two of the most-significantly PD-L1-associated genes. Consistently, a recent publication also reported that RBMS1 is a critical regulator in PD-L1 expression and tumor immunity. RBMS1 directly stabilizes the mRNA of B4GALT1, a glycosyltransferase that promotes PD-L1 maturation and stabilization via mediating the glycosylation of PD-L1 in breast cancer [18]. Here, we also observed a strong correlation between RBMS1 and PD-L1, not only in breast cancer, but also in multiple cancers. This suggests that RBMS1 may have a broad effect on PD-L1 across different types of cancer. It also supports the idea that our identified PD-L1-associated RBPs have important roles in the regulation of PD-L1, which should be further investigated.

Mature miRNAs induce the silencing of the target mRNA through an imperfect base-pairing, usually within the 3′-UTR [19]. Besides the important role of miRNAs in carcinogenesis, miRNAs have also gained attention as tools and targets for novel therapeutic approaches [20]. Although miRNAs have been extensively studied in cancer, there has been a lack of comprehensive analysis on the miRNAs that regulate PD-L1. In this manuscript, we identified 38 PD-L1-associated miRNAs, some of which have already been published. For example, miRNA-497-5p directly targets PD-L1 and downregulates its expression in clear cell renal cell carcinoma [21]. In addition, miRNA-93-5p downregulates PD-L1 in colorectal cancer [22]. However, these two publications focused on single cancers. In our analysis, the majority of the identified miRNAs are associated with multiple cancers, suggesting the need for further systematic exploration.

N6-methyladenosine (m6A) is the most-abundant internal modification in mRNA molecules. This modification is reversible and dynamically regulated by proteins known as “Writer”, “Eraser”, and “Reader” [23]. The PD-L1 mRNA is also modified by m6A, and its regulators have been extensively studied [24,25]. The level of PD-L1 mRNA is closely linked with ALKBH5, an Eraser of m6A modifications [25]. The m6A of PD-L1 is recognized by YTHDF2, which is a Reader of m6A [25]. Besides the reported m6A regulators, we also uncovered several new PD-L1-associated m6A regulators. Among them, ALKBH3 shows a significant association with PD-L1 in various types of cancer. Therefore, a thorough investigation of the precise regulation of PD-L1 m6A modification is warranted. Although we verified that the PD-L1-3′-UTR could promote PD-L1 expression in SUM-159 cells, the 3′-UTR regulators (RBP, miRNA, and m6A regulators) based on data mining have not been verified by wet experiments. Finally, we constructed a model using four genes, RBMS1, ZC3HAV1, QKI, and RBM38, to calculate the Tumor Immune Dysfunction and Exclusion (TIDE) score. In the TCGA cohort analysis, the TIDE score based on these four genes showed a good correlation with the patient’s response to ICI. Therefore, the four-gene TIDE score should be further validated in ICI-treated patients and might be beneficial to predict ICI treatment and prognosis.

In summary, we presented compelling evidence highlighting the crucial role of the 3′-UTR in the regulation of PD-L1. More importantly, by a systematic analysis, we identified several novel regulators of PD-L1. These findings contribute to a comprehensive understanding of the post-transcriptional regulation of PD-L1 through the 3′-UTR.

## Data Availability

The data that support the findings of this study are available from the corresponding author upon reasonable request.

## Supplementary Materials

The following Supporting Information can be downloaded at: https://www.mdpi.com/article/10.3390/genes14081620/s1, Figure S1: LASSO-Cox regression model building based on 43 RBP genes and 21 m6A-related genes. (A) Partial likelihood deviance as a function of regularization parameter λ in the TCGA dataset. Dotted vertical lines are drawn at the value with the minimum criteria and 1 standard error of the minimum criteria. (B) Curves represent the regularization paths of the LASSO coefficients. (C) The forest plot for univariate Cox regression analysis identified six genes associated with OS. Table S1: Multifactorial Cox regression results of four genes (RBMS1, ZC3HAV1, QKI, RBM38).
