# Supplementary figures and images for: The 3′ Non-Coding Sequence Negatively Regulates PD-L1 Expression, and Its Regulators Are Systematically Identified in Pan-Cancer
Source: Genes (Basel). 2023 Aug 13;14(8):1620. doi: 10.3390/genes14081620 (PMC10454350; doi:10.3390/genes14081620)

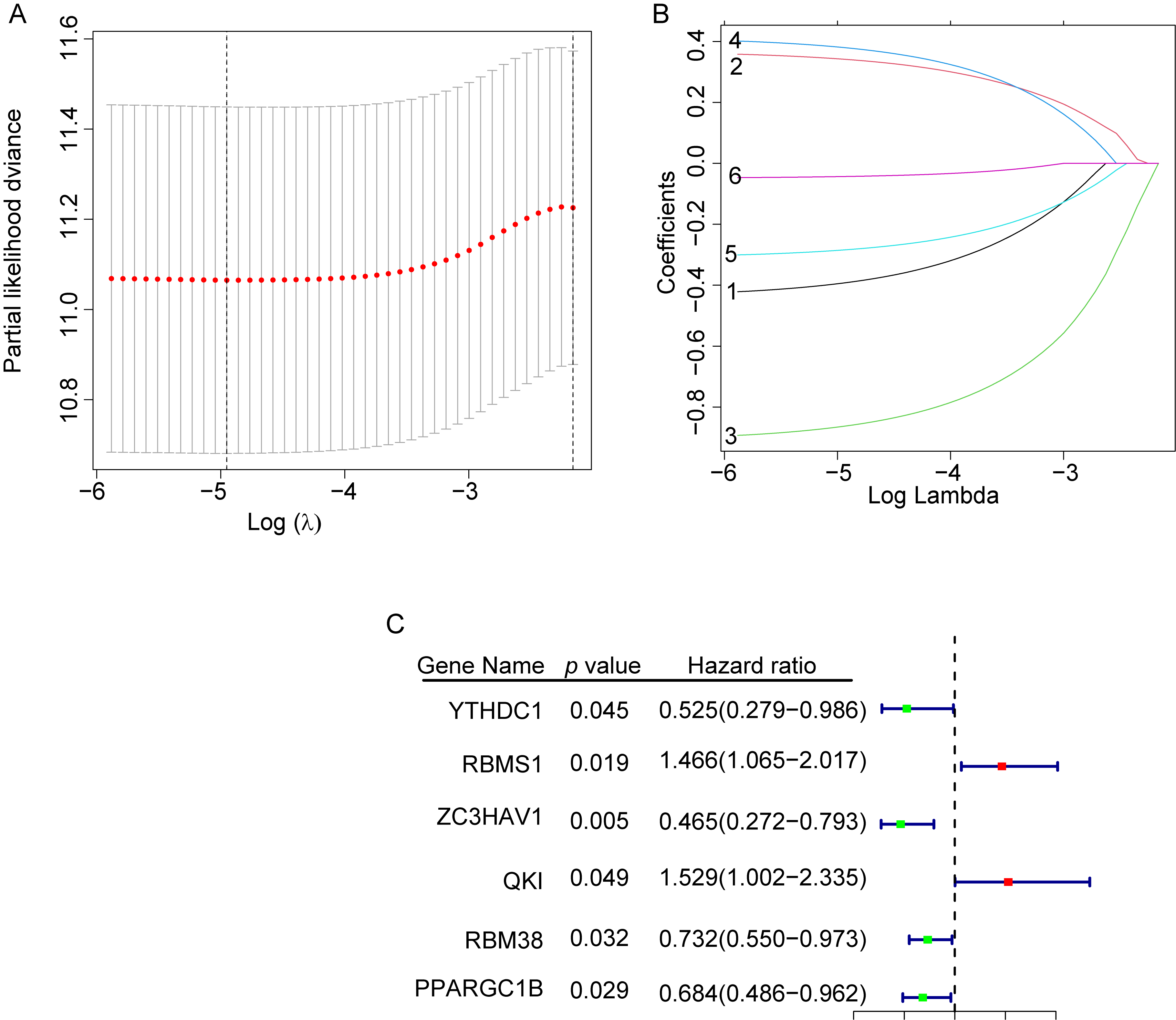

Supplement: Supplementary file 1 [file genes-14-01620-s001.zip › Fig S1.tif]
